# Primary care quality in Vietnam: Perceptions and opinions of primary care physicians in commune health centers – a mixed-methods study

**DOI:** 10.1371/journal.pone.0241311

**Published:** 2020-10-29

**Authors:** Nguyen Thi Hoa, Anselme Derese, Wim Peersman, Jeffrey F. Markuns, Sara Willems, Nguyen Minh Tam

**Affiliations:** 1 Department of Family Medicine, Hue University of Medicine and Pharmacy, Hue University, Hue, Vietnam; 2 Department of Public Health and Primary Care, Ghent University, Gent, Belgium; 3 Research Group Social and Community Work, Odisee University College, Brussel, Belgium; 4 Department of Rehabilitation Sciences, Ghent University, Gent, Belgium; 5 Global Health Collaborative, Department of Family Medicine, Boston University, Boston, MA, United States of America; Institute of Mental Health, SINGAPORE

## Abstract

**Introduction:**

Measuring the performance of a primary care system is one of the very first steps to find out whether there is room for improvement. To obtain an objective and comprehensive view, this measurement should come from both the supply and demand sides of the system. Patients’ experiences of primary care have been studied around the world, but much less energy has been invested in researching providers’ perspectives. This research aims to explore how primary care physicians working at commune health centers in Vietnam evaluate their performance and their opinions on how to improve the quality of primary care services.

**Materials and methods:**

First, a quantitative study was conducted using the validated Vietnamese PCAT questionnaire—provider expanded version (VN PCAT PE) targeting all primary care physicians (PCPs) working at commune health centers in a province of Central Vietnam. Next, a qualitative study was carried out, consisting of in-depth interviews with PCPs, to better understand the results of the quantitative survey and gain insight on barriers of primary care services and how to overcome them.

**Results:**

In the quantitative portion of our study, 150 PCPs rated the quality of ongoing care and first contact in CHCs as the best (3.09 and 3.11 out of 4, respectively), and coordination as the worst performing core domain (2.53). Twenty-two PCPs also participated in our qualitative research. In regards to challenges that primary care physicians face during their daily practice, three central themes emerged: 1) patient factors such as client attitude and knowledge, 2) provider factors such as the burden of administrative work and lack of training opportunities, and 3) contextual factors such as low income and lack of resources including medicines and diagnostics. Participants recommended more health promotion campaigns in the media, increasing the number of services available at CHCs (such as being able to take blood samples), reducing the workload related to administration for CHC leaders, greater government subsidies, and providing more training courses for PCPs.

**Conclusions:**

Findings from this study offer a valuable view from the supply-side of the primary care system, specifically those who directly deliver primary care services. Along with the earlier study on consumers’ evaluation of the Vietnamese primary care system, and literature from other low and middle-income countries, these findings offer emerging evidence for policymakers to improve the quality of primary care in Vietnam.

## Introduction

One of the United Nations’ Sustainable Development Goals is to achieve universal health coverage, defined as financial risk protection, access to quality essential health care services and access to safe, effective, high-quality and affordable essential medicines and vaccines for all [1]. To achieve this goal, investment to improve the quality of primary care would be one of the most vital components for all countries. Recently, in the new Astana Declaration on Primary Health Care, the commitment of states and governments has been reaffirmed toward the establishment of “a sustainable primary health care” as well as the improvement of “the capacity and infrastructure for primary care—the first contact with health services” [2].

Measuring the performance of primary care service delivery is one of the very first critical steps in identifying areas for improvement [3], and multiple efforts have been underway over the last few decades. Examples include the Primary Health Care Vital Signs Profile from the Primary Health Care Performance Initiative utilizing a range of existing global and country level data to present a system-level view of primary health care service delivery, as well as the Primary Care Evaluation Tool from the WHO Regional Office for Europe consisting of three separate questionnaires: one on the situation of primary care policies and structures at the national level, one for primary care physicians and one for patients [4, 5]. Looking more specifically at delivery of primary health care services at the point of care, the Primary Care Assessment Tool (PCAT) from Johns Hopkins Primary Care Policy Center is a long standing tool based on core primary care principles, used globally and validated in multiple countries. This survey tool includes four versions: an adult consumer survey, a child consumer survey, a provider survey and a facility survey [6].

Various studies around the world have explored patients’ experiences of primary care, and have often revealed systemic problems that affect quality and efficiency [7, 8]. To obtain an objective and comprehensive view of this service delivery, evaluation should come from both sides of the system: users (patients) on the demand-side, and providers (health care professionals) and health care managers on the supply-side. A recent study in Vietnam used a validated version of the adult consumer version of the PCAT to survey residents on their experiences of different primary health care facilities [9]. In contrast, relatively little energy has been invested in learning how primary care physicians perceive the status of the services they are providing and the environment they are working in. A recent South African study highlighted that there was a significant gap between clients’ experience with primary care and what managers and providers thought they were delivering [10].

The current health system in Vietnam is a mixed public-private system, in which the public system plays a critical role in preventive and curative care for the population nationwide. The public health care system is a four-tier system: central, provincial, district, and commune. The central and provincial levels are classified as tertiary and secondary care with specialised health care professionals, while district and commune levels deliver primary health care services. Being the foundation of the health system, primary care is considered of great significance in the national health program of Vietnam [11].

In Vietnam, primary care physicians (PCPs) often work at commune health centers (CHCs), together with nurses, midwives, pharmacists, and others. A PCP is a general doctor with or without post-graduate speciality training in family medicine or other specialities. They often work as the head of CHCs and also provide clinical services such as examination and treatment of patients. The widespread network of CHCs across the country functions as a gatekeeping mechanism to the health care system. However, despite the Ministry of Health’s efforts to improve primary care quality in recent years [12–18], patients continue to bypass these facilities and choose to consult secondary or tertiary levels of care directly, presumably because they expect to obtain higher service quality from those levels, even if at a higher out-of-pocket cost. Interestingly, our own prior work has explored how patients experience primary care at various health care facilities, and noted primary care quality was rated highest at CHCs [9].

We conducted this study to explore how primary care physicians working at CHCs in Vietnam evaluate their own performance and what they perceive can be done to improve primary care and strengthen their role as the primary entry point to the health care system.

Specifically, this study sought to answer the following questions:

1. How do primary care physicians working at commune health centers evaluate the performance of their services?
2. What are the barriers to providing high quality primary care services according to the primary care physicians working at commune health centers, and what do they recommend to overcome those barriers?

## Materials and methods

**Fig 1:**
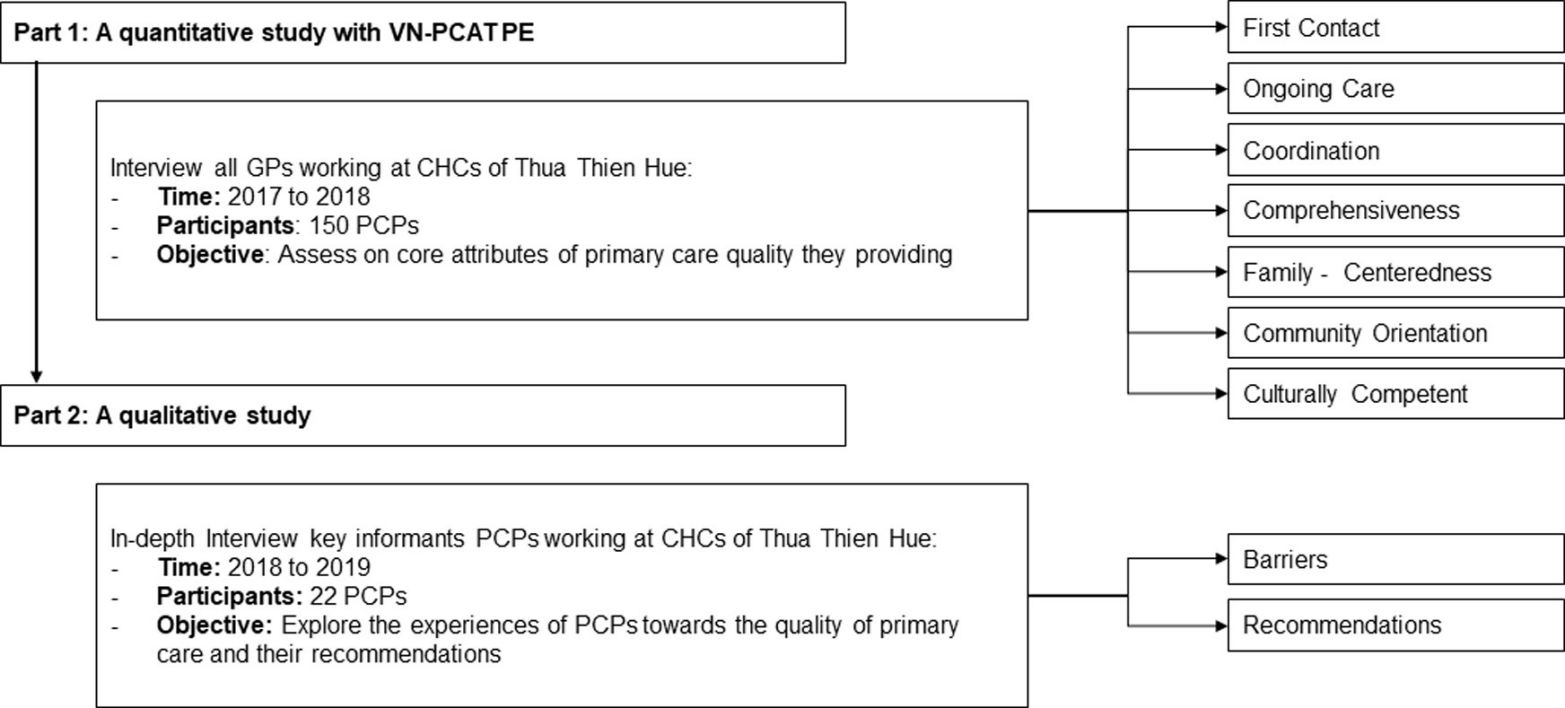
Study design.

We used a mixed methodology in this study (Fig 1). First of all, a quantitative survey was conducted, using the validated Vietnamese PCAT Questionnaire—Provider Expanded version (VN PCAT-PE) [19] among primary care physicians (PCPs) working at CHCs in a chosen province. Next, a qualitative study was carried out, consisting of in-depth interviews with PCPs, to better understand the results of the quantitative survey and gain insight on how to improve the quality of primary care services.

### Study context

This study was carried out in Thua Thien Hue, a coastal province in Central Vietnam with a population density of 235 persons/km² [20]. The health care system in Thua Thien Hue is similar to other provinces throughout Vietnam, typically with a district hospital surrounded by a network of CHCs at the primary care level in each district. In addition, there are three general hospitals, seven specialist hospitals and one central hospital in the state-run system located in Thua Thien Hue [21].

In total there are 152 CHCs in the province. In general, each CHC is equipped with a PCP as head of the health care team, an assistant doctor, a nurse, a midwife, and a pharmacist [22]. In some districts, where there is a lack of PCPs, their positions may be filled by an assistant doctor, a traditional medicine doctor, an assistant traditional medicine doctor or a preventive doctor. Based on the needs, the district health authorities may alter the composition of the local primary health care team. In Hue City, for instance, some PCPs are asked to also take charge of a neighbouring CHC without a PCP. These PCPs work three days at their own CHC and two days at the alternate CHC. In other districts, the non-PCP CHC examination and treatment are carried out by assistant doctors. In some CHCs located in mountainous areas, by contrast, there may be more than one doctor at the CHCs. Most of these have recently been upgraded from assistant doctors to general doctors after completing an additional 4-year training program.

### Sampling

#### Quantitative study

This census study surveyed all PCPs working at commune health centers in Thua Thien Hue Province who consented to participate in the study. These PCPs had at least one-year of experience as a PCP at a CHC.

#### Qualitative study

For the qualitative portion, it was required that participants had at least one-year of experience as a PCP at a CHC. We planned to carry out in-depth interviews until information saturation was achieved. The anticipated number of interviewees was estimated to be 20 to 25. To achieve representativeness, we tried to balance our purposive selection of participants by location of workplaces (urban or rural), number of years in practice, and whether or not they had completed post-graduate training using data from our quantitative study surveying all PCPs working at CHCs in Thua Thien Hue province.

**Fig 2:**
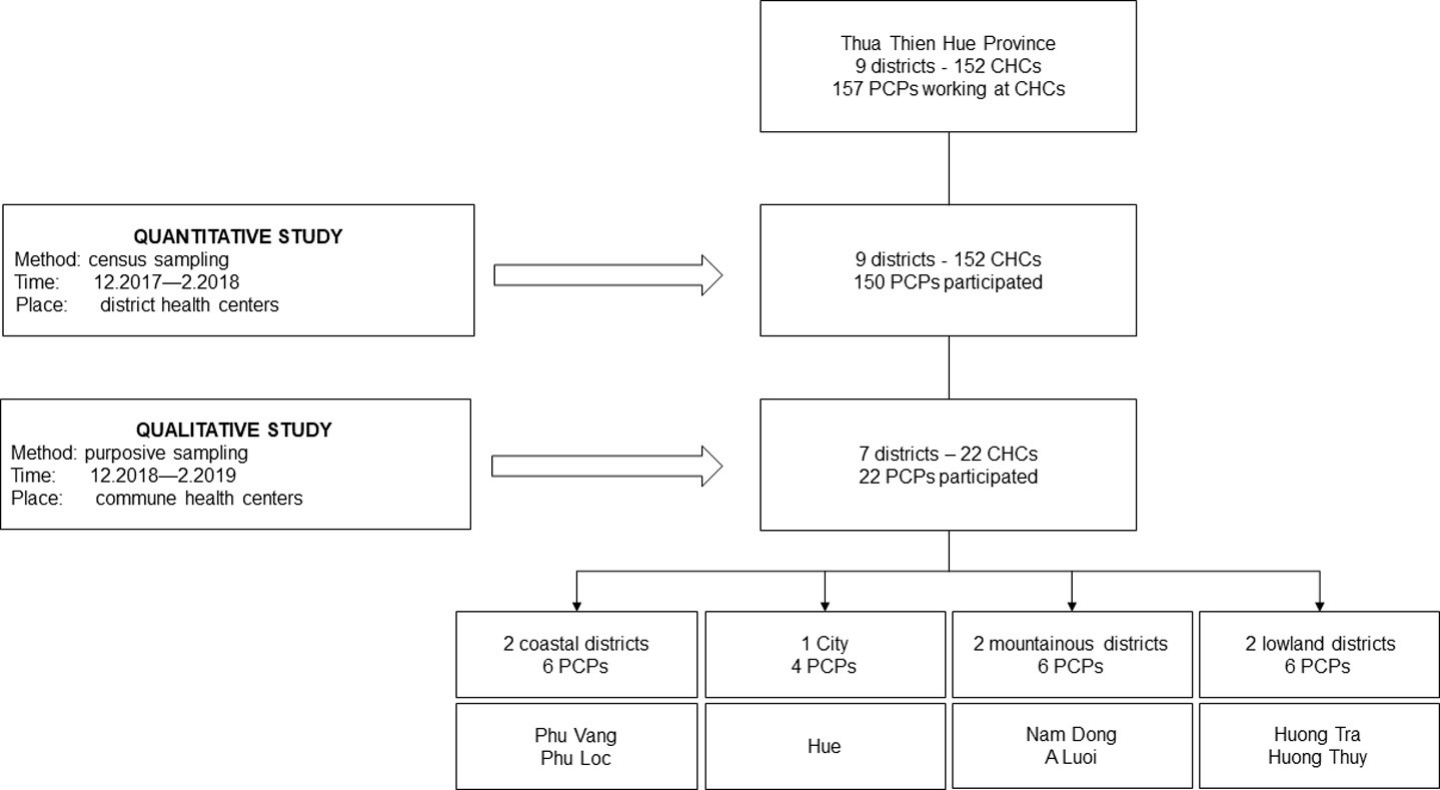
Sampling procedure.

We believe the sample for the quantitative portion of this study can be considered representative of PCPs working in CHCs throughout the country, and while not designed to be generalizable, we expect results of the qualitative portion of this study to be transferable to the experience of many other PCPs (Fig 2).

### Data collection

#### Quantitative study

An adaptation of the Provider Expanded version of the Primary Care Assessment Tool (PCAT-PE), originally developed at Johns Hopkins University, was employed as the major investigation instrument in the present research. The validated version of this tool for Vietnam (VN PCAT-PE) contains 116 items on six scales representing four core primary care domains, namely 1) first contact, 2) ongoing care, 3) coordination, and 4) comprehensiveness of services; three additional scales representing three derivative domains of 1) family centeredness, 2) community orientation, and 3) cultural competence [23]. The process of recording and calculating the sum mean score of domains as well as subdomains of primary care strictly complied with the guidelines in the PCAT manual [24].

The questionnaire, which took 30 to 45 minutes to self-complete, was delivered at the end of a staff meeting held monthly at each district health center. If a PCP was absent at that meeting, he or she was contacted for an appointment at their CHC, and otherwise excluded from our research after three unsuccessful attempts.

Prior to the study, participants received a full explanation of its content and purpose, then signed a consent form if they agreed to participate. 5 USD was offered to each participant as a small token of appreciation. Quantitative data was collected from December 2017 to February 2018.

#### Qualitative study

An interview guide was developed to explore the views of interviewees, and the core questions asked were as follows (S1 Appendix):

1. What do you think about primary care quality?
2. What are the barriers/challenges to primary care quality?
3. What should be done to improve the current situation of primary care?

Interviews were conducted in Vietnamese by two research assistants, who both held Master’s degrees in public health. The interview location was privacy-assured, mostly at their CHCs, and the time was suitable for the participants. Each interview lasted 40 to 60 minutes and was tape-recorded with the consent of the participant; notes were taken in Vietnamese and the transcripts were completed by the interviewers. The whole process was closely supervised by NTH, the principal investigator, with regular audiotape and transcription review, and revision of the interview guide if needed during the data collection period. 10 USD was offered to each interviewee as a token of appreciation. Qualitative data was collected from December 2018 to February 2019.

Ethical approval for the study was granted from the Scientific Committee of Hue University of Medicine and Pharmacy on March 20^th^, 2015. Written informed consents from all participants were obtained prior to interviews.

### Data analysis

#### Quantitative study

Quantitative data were analysed with the SPSS software version 24.0.

#### Qualitative study

Data were analysed using a thematic framework [25, 26], all the interview data were coded and analysed according to the seven stages of this method: transcriptions, familiarisation with the interviews, coding, developing a working analytical framework, applying the analytical framework, charting data into the framework matrix, and finally, NTH interpreting the data. All codes and themes were presented to other research team members (AD, WP, JM, SW and NMT) for discussions through emails on data interpretation results and key findings. NVivo12 (QSR International, www.qsrinternational.com) was used to code all transcripts. The checklist for consolidated criteria (COREQ Checklist) for reporting qualitative research was utilised to report the research process and results [27].

## Results

### Characteristics of participants

150 out of a total of 157 PCPs working at CHCs participated in our quantitative study. Among them, 22 PCPs continued to take part in our in-depth interviews. Tables 1 and 2 show the characteristics of the participants and their workplaces. In the sample for the quantitative study, there were about twice as many male doctors as female ones; three-fifths of these doctors had been practising for 20 years or more. Although CHCs provide care for patients of all ages, most of their patients were adults. Consistent with national data on the medical workforce, 38.9% of PCPs working at CHCs were female [22].

**Table 1:** Characteristics of study population: Primary care physicians.

Characteristics	Quantitative study (N = 150)	Qualitative study (N = 22)
	n (%)	n (%)
**Gender**		
Female	52 (34.7)	13(59.1)
Male	98 (65.3)	9 (40.9)
**Age**	Mean 46.2, SD 7.85, Range (29–60)	Mean 47.3, SD 8.24, Range (30–54)
29 to 39-year-old	33 (22.0)	5 (22.7)
40 to 50-year-old	62 (41.3)	4 (18.2)
51 to 60-year-old	55 (36.7)	13 (59.1)
**Number of years in practice**	Mean 18.32, SD 9.3, Range (1–35)	Mean 21.5, SD 9.4, Range (3–32)
less than 10 years	35 (23.3)	4 (18.2)
10 to 19 years	24 (16)	2 (9.1)
20 to 29 years	83 (55.3)	13 (59.1)
30 years and more	8 (5.3)	3 (13.6)

**Table 2:** Characteristics of study population: Health facilities (N = 150).

Characteristics	Mean (SD)	Range
**Number of consultations per day**	28.7 (14.2)	(5–95)
**Percentage of consultations by age**		
0–6 years old	20.0 (14.2)	(0–100)
7–16 years old	15.2(8.5)	(0:50)
17–59 years old	34.9 (18.2)	(0:85)
60–80 years old	20.5 (11.2)	(0:60)
>80 years old	10.1 (8.1)	(0:50)
**Percentage of chronic patients**	**n (%)**	
Less than 20%	86 (62.8)	
From 20 to 40%	37 (27.0)	
From 41 to 60%	10 (7.3)	
More than 60%	4 (2.9)	

### Primary care assessment from the physicians’ view

**Fig 3:**
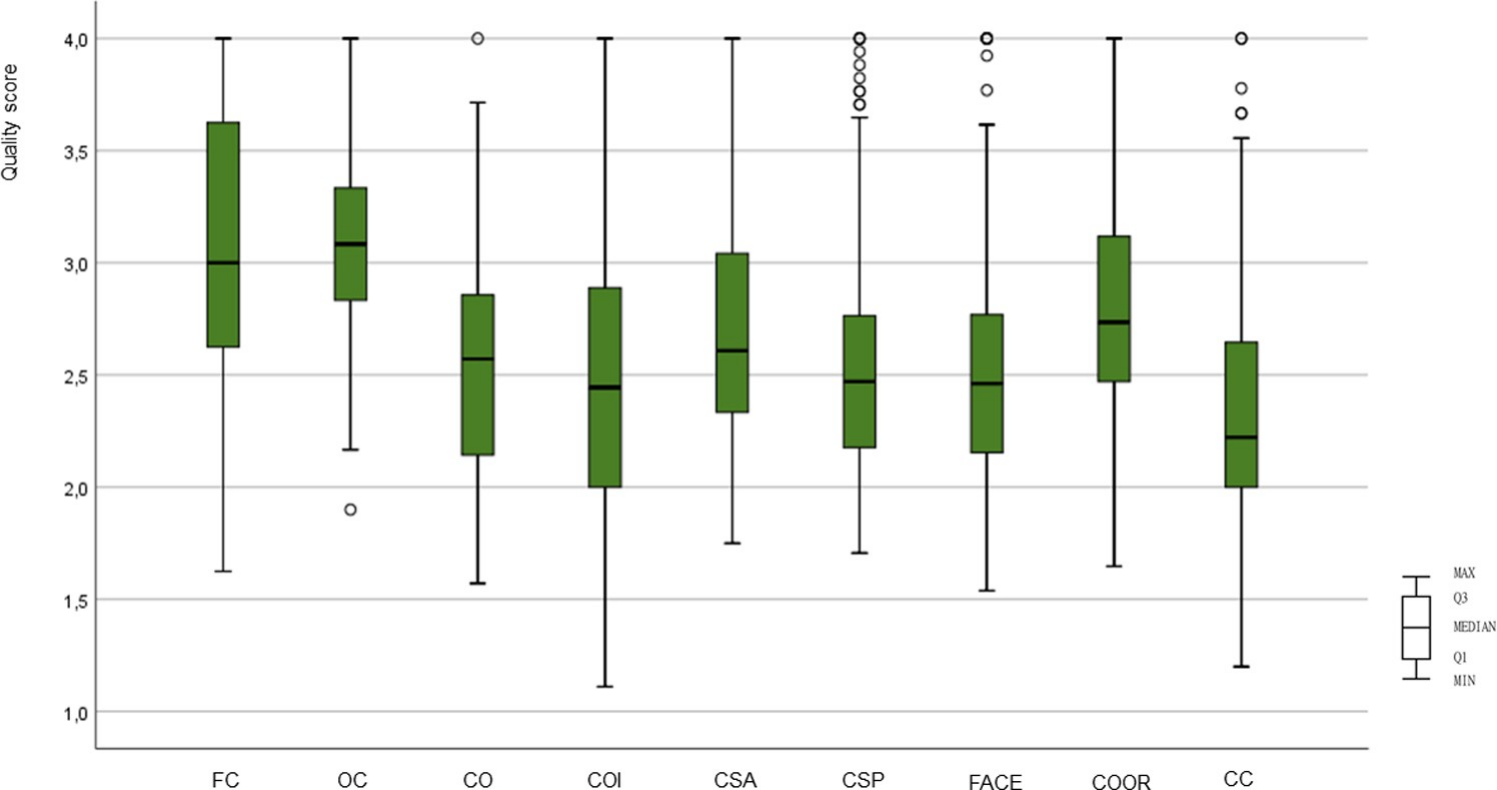
Primary care assessment from physicians’ perspectives (PCAT score) (N = 150). FC: First Contact; OC: Ongoing care; CO: Coordination; COI: Coordination (information system); CSA: Comprehensiveness (Services available); CSP: Comprehensiveness (Services provided); FACE: Family-Centeredness; COOR: Community Orientation; CC: Culturally Competent.

PCPs working at CHCs rated the quality of primary care as 16.34 (maximum potential score 24) on the PCAT score and 23.95 (maximum potential score of 36) on the PCAT expanded score. (Table 3, visualised by Fig 3). Ongoing Care and First Contact were the primary care attributes that PCPs rated the highest. Coordination was rated as having the worst performance amongst the core domains. With regards to three derivative domains, Cultural Competency scored lowest in quality of performance.

**Table 3:** Primary care assessment from physicians’ perspectives (PCAT score) (N = 150).

Domain	Mean	SD
First Contact	3.09	0.60
Ongoing Care	3.11	0.44
Coordination	2.53	0.51
Coordination (Information system)	2.44	0.64
Comprehensiveness (Services available)	2.70	0.49
Comprehensiveness (Services provided)	2.58	0.54
Family-Centeredness	2.50	0.52
Community Orientation	2.83	0.51
Culturally Competent	2.32	0.57
PCAT score	16.34	2.32
PCAT expanded score	23.95	3.41

### Challenges of primary care quality and recommendations from primary care physicians

An overview of the results of the qualitative research can be found in Table 4. Challenges perceived by primary care physicians and their recommendations were categorized into three major factors: patients, providers themselves and contextual factors. Several suggestions had been raised by the respondents in order to improve the quality of primary care and recruit patients back to their health care facilities.

**Table 4:** Thematic matrix.

Category	Patient factors	Provider factors	Contextual factors
**1. Challenges of primary care quality**	• Perception of passing by the grassroots level to upper level care due to better technology, going to a private clinic, or simply presenting to a pharmacy without a prescription.	• Only one doctor at CHCs, responsible for both administrative and clinical work	• Administrative burden
			• Do not have the budget for daily repairs for equipment.
			• Lack of medication for non-communicable diseases.
			• Reimbursement process from the health insurance company is complicated
	• Low level of patient knowledge in some rural mountainous areas		
			• Low income compared to other employment options
**2. Recommendations for improving primary care quality**	• Enact media campaigns for patients about health promotion and services available at CHCs	• Additional PCP or reduce the workload related to administration for CHC leaders	• Ensure an adequate supply of medication
			• Offer more lab tests such as blood glucose measurement
			• Have CHCs collect blood samples and deliver them to the nearby district health center for results.
		• Provide frequent short training courses to update clinical knowledge	
			• Provide greater subsidy from the government for PCPs working at CHCs.

#### Challenges of primary care quality

*Patient factors*. Some factors related to patients’ knowledge and perceptions were considered barriers to primary care at the CHCs. The bypassing behaviour of skipping primary care and moving to an upper level was still prevalent and believed to be due to superior technology at the upper levels. In addition, it was believed patients go to pharmacies and consult pharmacists instead of physicians because patients perceive it to be faster and more convenient.

*“…The most common problem is that pharmacies sell prescription medications without a doctor’s prescription*. *Due to this so-called convenience and wrong personal perception*, *people do not come to us*, *nor private clinics*, *as they prefer to buy medications at the pharmacy*. *When the patient’s condition does not improve*, *they will then visit the CHCs…" P14*

Moreover, PCPs encountered difficulties in providing health care for people in rural mountainous areas, citing a perception that most local residents focus on religious beliefs and divine healing powers or alternative treatments rather than consulting doctors or health experts.

*"…When older people have health problems such as breathing difficulties or arthralgia*, *some of them choose their own method of treatment over seeing doctors in the first place*. *They would offer rice*, *chicken*, *and money to the priests to hold a religious ceremony…" P11*

*Provider factors*. As head of the CHC, most of the PCPs expressed that they had a heavy workload. They were responsible for both administrative and clinical work at the CHCs. Furthermore, PCPs were not allowed to take a day off after a 24-hour duty shift, unlike other health care staff, because they were the only ones who could examine and treat patients. Specifically, due to a lack of human resources, CHCs in Hue city were not all staffed with PCPs; hence, some PCPs were asked to also be responsible for a neighbouring CHC that lacked a qualified PCP.

*"…I am myself a general doctor*, *and I see patients*, *do ultrasound tests*, *carry out procedures such as suturing a wound in need*, *attend meetings*, *etc*. *I cannot grasp the whole of this job…" P13*

*"…Currently*, *the number of visits is decreasing because I am studying and working at the same time*. *Moreover*, *I have to oversee two other CHCs resulting in less time for one single CHC*, *which leads to limited contact with patients…" P18*

*Contextual factors*. Apart from direct clinical duties, PCPs reported also being responsible for implementing around 18 national vertical programmes in their corresponding communities such as those of immunisation, HIV, dengue fever, and mental health. The surveyed PCPs complained that there was more paperwork than they were able to handle and that these programme reports stole a lot of their time from patient care.

*"…Too much work has to be done*, *with various reports per month, including the malaria programme and HIV programme*. *Tasks just disperse at a CHC*, *where each programme has to be reported in separate notebooks…" P04*

Additionally, the PCPs in this study regarded the lack of essential equipment and medication as one of the limiting factors. Despite governmental and NGO project support, some CHCs still lacked necessary equipment such as ECG and ultrasound machines, and those with such equipment lacked a regular maintenance budget. CHC formularies did not include medicines sufficient for treating all types of disease that the PCPs thought they could manage at the primary care level, especially non-communicable diseases such as diabetes and hypertension.

*"…The medication list doesn't meet the needs of patients with chronic diseases such as hypertension*, *cardiovascular disease*, *hyperlipidaemia and asthma*. *Therefore*, *more and more patients tend to take other routes of medical treatment…" P02*

The PCPs also reported on so-called “complicated” reimbursement by health insurance; recent changes in the social health insurance policy like digitising reports or audits requiring the CHC staff to frequently change the report form or diagnosis and procedure terms, as well as update new software. If anything went wrong with these reports, they would not be reimbursed by the social health insurance.

*"…The health insurance company has changed the rules all too often*. *Last month*, *they announced a new software update*. *They have also released more limitations to prescription forms*, *payment vouchers*, *complicated reports*, *types and quantities of medicine…" P18*

Lastly, the PCPs in this study lamented their low salary compared to other workplaces. Therefore, outside of their regular business hours some also engaged in other jobs such as farming or providing health care services at home for the elderly. Home care, nevertheless, was not considered an authorized activity for them to receive payment directly from patients.

*"…Yes*, *we do provide home care visits for the elderly*. *It is a duty to take care of the elderly without expecting any payment in return…" P10*

#### Recommendations

Several suggestions were put forward by the research participants in order to improve the quality of primary care and attract patients back to their health care facilities.

*Patient factors*. To raise patients’ awareness of primary health care and the related services available at CHCs, the PCPs suggested that there should be more mass media campaigns for health promotion and to increase awareness of health care services accessible to patients and local inhabitants. Relevant health stakeholders should support this kind of activity with advertising brochures or television and radio commercials.

*"…Let us coordinate with the Department of Information and Communications to spread our messages through television or radio*. *It should also work better if we combine the medical part with the communication activities of the women's union…" P01*

*Provider factors*. The surveyed PCPs asked for one more GP or preventive doctor if possible, to reduce the workload of the CHCs’ leaders and to have a doctor always available for patient care. For the health care providers themselves, PCPs cited the need for frequent knowledge updates as the most important recommendation. PCPs preferred intensive training courses that lasted from only a few days up to three months, focused on common and chronic diseases. This frequent type of training would not only prove suitable for the health care staff’s level of expertise but also help minimize time away from work at the CHCs given primary health care workforce shortages. Some PCPs also mentioned the need for training in communication skills and professionalism for all health care providers at CHCs.

*“…We should also train the medical staff how to communicate effectively with different subjects such as the elderly or children*, *how to solve problems*, *and how to practise mindfulness…” P21*

*Contextual factors*. PCPs recommended having adequate medication for common health problems at CHCs, especially for chronic diseases which require monthly visits. However, they recommended that CHCs should also have more lab tests such as glucose or lipid measurement so that patients would not have to go to upper levels just for these tests. One solution to this issue was sending patients’ blood samples, taken at CHCs, to the district hospital for analysis. The results would be sent back to the original CHCs for the PCPs’ reference.

*"…To illustrate*, *diabetes patients should have a glucose blood test frequently during their treatment period*. *Unfortunately*, *we don't do it here*, *so they tend to go to a hospital more fully equipped with testing methods and medicine*. *We cannot manage those patients…" P12*

One PCP suggested combining nearby CHCs into a larger CHC to solve the shortage of human resources and equipment. *“*…*If we cannot have more staff or equipment*, *we can combine two or three CHCs in an area to make one big CHC*. *This new center would serve the local population better as it would have enough doctors and equipment* …*” P18*

Finally, most PCPs raised concern that there is a need for greater subsidies from the government for doctors and other CHC health care staff, which would then aid in attracting more young doctors to work there.

*“…The authorities should issue more specific policies to support the medical staff at CHCs*. *Otherwise*, *it will be more difficult to engage young doctors because no one wants to work on a low salary*, *from which they cannot pay their household expenses …” P16*

## Discussion

The objective of this mixed-methods study was to reveal Thua Thien Hue PCPs’ perceptions of the quality of their services. PCPs assessed the quality of their primary care and identified key challenges to primary care before suggesting solutions to improve the situation.

### Strong and weak points of the CHCs

As CHCs are located in every commune throughout the country, PCPs felt they could manage local health care well and understand patients’ cultures and social context, which was a fundamental element of continuous care (ongoing care): a strong patient-doctor relationship built over time. With a known and trusted doctor and without obstacles to communication, good continuous care could lead to positive effects on treatment outcome and health care quality [28]. PCPs in our survey reaffirmed that Ongoing Care and First Contact were the best primary care attributes they performed. On the other hand, our previous PCAT study of consumer experiences showed that CHCs were the most common choice of respondents as a usual source of primary care. In that study, consumers gave CHCs the highest scores for Ongoing Care and First-Contact utilization and the next highest score for First-Contact access in comparison with other types of health care facilities [9]. The consistency of this assessment from both the supply- and demand-sides implies that CHCs are a reliable first-contact point for patients at the primary care level.

PCPs and consumers also share similar assessments of Coordination as the worst performing primary care core domain [9]. Some explanations for this may be that the two—way referral and counter-referral system between the CHCs and upper level facilities still does not exist in Vietnam, and the connection between the private and public sectors is also still weak [29]. The transformation from a paper-based system of the Ministry of Health in Vietnam to an electronic-based one could help to improve the coordination between health care professionals at different levels, leading to better patient management and follow-up, especially for those with chronic diseases [30]. A Chinese study indicated that both primary care physicians and patients regarded Coordination as the weakest dimension of primary care service capacity [31]. Even in high-income countries, a high percentage of primary care physicians reported that they failed to routinely receive timely information from specialists or hospitals [32]. Coordination of care was also identified as the weakest dimension of family medicine in a study among 34 countries by Pavlič in 2015 [33]. Improving the cooperation and interaction between all levels of care would therefore be crucial for every country to achieve better management at the primary care level.

### Challenges and recommendations for primary care in Vietnam

Like in many other developing countries, the lack of essential medication and equipment were identified by doctors in our study as main factors inhibiting high-quality care at the primary care level. Beyond that, there were three central themes of challenges that primary care doctors faced in their daily practice.

The first major obstacle was patients bypassing primary care and choosing to be treated in a more fully equipped hospital at a higher level, or relying solely on pharmacists’ advice, or even merely self-treatment. In an earlier consumer survey among 1662 adults living in Central Vietnam, 15.3% and 11.8% of residents utilized high–level hospitals such as provincial or central hospitals and pharmacies (in that order) as sources of primary care [9]. Together with policy changes restricting medication sales without a prescription at pharmacies, primary care doctors suggested that media campaigns on health promotion and CHC services should be launched more frequently. This is consistent with international studies suggesting that because of their wide reach, appeal, and cost-effectiveness, media campaigns have been major tools in health promotion and disease prevention and could result in a modest increase in utilization of health services [34, 35]. With regards to lab tests such as glucose measurement for diabetes management, an interesting recommendation was raised: CHCs could take blood samples and have them delivered to the nearby district health center for analysis. This is also a common practice in Europe and the U.S where many individual primary care practices have laboratory samples analyzed off-site [36–38]. Using this approach, patients would not have to visit upper levels just for more lab tests, offering a partial solution to the current overcrowding at upper levels. The participants also stressed the burden of administrative work, as CHCs were typically equipped with only one PCP, responsible for both administration and clinical care. Doctors in city areas often were in charge of two or even three CHCs. This problem is also common elsewhere in the world [39, 40]. A study conducted by the US Commonwealth Fund in 11 countries indicated that the time required for administrative and other tasks besides patient contact was seen as a significant drawback. Also in Germany, the majority of primary care physicians indicated that the time needed for those activities was “very problematic” [39]. In South Africa a qualitative study with key leaders of the district health system also shared the concern that administrative functions might well overwhelm the clinical role of family physicians [40]. Mitigating CHC leaders’ paperwork load was considered by providers as a crucial solution to improving the quality of care by making more time available for patients.

Last but not least, the low income compared with other workplaces was cited as one of the main barriers to providing quality primary care. Poor career benefits such as low income and lack of training opportunities were perceived as a significant obstacle to attracting young physicians to join the primary care system. This issue has been reported in previous studies in Vietnam and other countries suggesting that barriers to recruitment and retention of health care workers at the primary care level may be due to both financial and non-financial factors such as inadequate training, unprofessional work environments, and insufficient remuneration [41–43]. Another study also found that opportunities to attend in-service training for doctors in rural Vietnam was limited due to the shortage of available health care workers to provide coverage [44]. In our study, the majority of PCPs expressed a need for postgraduate training, specifically short-term courses to reorient and sharpen the existing workforce’s skills at the primary care level. As was highlighted in a previous review of the roles of, and training for, primary care doctors in China, India, Brazil and South Africa, quality of primary health care is clearly seen as crucial to obtain the population’s trust in these services, and gains in health outcomes. Doctors with postgraduate training in family medicine were noted to play an indispensable role in ensuring this quality as part of a broader primary health care team [45].

### Limitations of the study

There were several limitations to this study. First, it was designed only to interview PCPs working in CHCs because CHCs are considered the major source of primary care in Vietnam. On the other hand, for a more diverse picture of primary care quality, future studies could investigate more thoroughly the various types of primary care providers working in the private sector or outpatient clinics of hospitals and other health care stakeholders such as policymakers and the local authorities, as well as the opinions of other health care staff working in CHCs. Also, due to limited resources, the study was carried out among CHC doctors in only one province of Central Vietnam, which might lead to some bias when generalising the study results to the national level. Given the same structure in the health care system of every province in Vietnam and the similar settings, working regulations, policies and investigations across all Vietnamese CHCs, it is nevertheless strongly believed that these research findings can still contribute reliable evidence to primary care more generally in Vietnam.

## Conclusions

As the very first research using mixed methods to survey health care providers’ perspectives of primary care quality in Vietnam, the present study offers a valuable view from the supply-side of the primary care system, from those who directly delivery primary care services. Along with the earlier study on consumers’ evaluation of the Vietnamese primary care system, and literature from other low and middle-income countries, these findings provide emerging evidence for policymakers to improve the quality of primary care in Vietnam. This paper also emphasizes the need for additional research on primary care provision and quality in Vietnam to strengthen the impetus for change.

## Supporting information

S1 Dataset Vietnam PCAT provider data. (SAV)

S1 Appendix Guidelines for in-depth interview. (DOCX)
