# Supplementary material for: Primary care quality in Vietnam: Perceptions and opinions of primary care physicians in commune health centers – a mixed-methods study
Source: PLoS One. 2020 Oct 29;15(10):e0241311. doi: 10.1371/journal.pone.0241311 (PMC7595414; doi:10.1371/journal.pone.0241311)
Supplement: S1 Appendix — (DOCX) [file pone.0241311.s002.docx]

**Guidelines for In-depth Interview**

**Primary care quality in Vietnam: Perceptions and opinions of primary care physicians in commune health centers – a mixed-methods study**

1. **General information**

- Name, Gender, Date of birth
- Number of years of practice
- Working place

1. **Primary care assessment**

- What do you think about primary care quality in general?
  - In your daily practice?
  - What are the strong and weak points?

1. **Barriers/Challenges to primary care quality**

- According to you, what are barriers in providing good primary care in your daily practice?
  - From the provider yourself (age/training/knowledge/gender…)
  - From the setting (salary/ health insurance company/scope of work…)
  - From the patients (knowledge/wealth level/health problems)

1. **Recommendation and needs**

- What should be done to improve the current situation of primary care? At what level? And what is the priority?
  - Policy
  - Staff capacity, number
  - For patients
  - Finance
  - Health insurance…
- As a primary care doctor, would you like to improve your practice by learning more? What do you want to learn?
  - Soft skills, teamwork?
  - Management skills?
  - Update knowledge
  - Manipulation procedure services…

Is there anything else that you want to discuss on this topic, but I have not mentioned above? Please discuss this with me. If not, thank you for your participation.
